# BOS-318 treatment enhances elexacaftor–tezacaftor–ivacaftor-mediated improvements in airway hydration and mucociliary transport

**DOI:** 10.1183/23120541.00445-2024

**Published:** 2025-02-25

**Authors:** Lisa E.J. Douglas, James A. Reihill, S. Lorraine Martin

**Affiliations:** School of Pharmacy, Queen's University Belfast, Belfast, UK

## Abstract

**Background:**

Cystic fibrosis transmembrane conductance regulator (CFTR) triple modulator therapy, elexacaftor–tezacaftor–ivacaftor (ETI) has transformed care for people with cystic fibrosis (CF) who have eligible mutations. It is, however, not curative. Response to treatment also varies and lung disease, although slowed, remains progressive. We have previously demonstrated inhibition of the epithelial sodium channel (ENaC) by selective furin inhibition to be an alternative, mutation-agnostic approach that can enhance airways hydration and restore mucociliary transport (MCT) in CF. Inhibition of furin therefore, offers a potential therapeutic strategy for those ineligible, intolerant or nonresponsive to ETI and may provide a further opportunity for clinical benefit for those currently treated with ETI. The aim of this study was to determine the impact of furin inhibition on ETI responses to assess its utility as an adjunct therapy.

**Methods:**

Differentiated primary CF human bronchial epithelial cells (HBECs) were treated with the highly selective furin inhibitor BOS-318 and with ETI. Ion channel function was measured using a 24-channel Transepithelial Current Clamp (TECC-24) system and airways surface hydration was investigated by measuring airway surface liquid (ASL) height and MCT rate.

**Results:**

The presence of BOS-318 had no effect on the ability of ETI to stimulate CFTR-mediated Cl^−^ secretion but contributed a reduced Na^+^ transport *via* robust inhibition of ENaC. This altered ion transport profile effected an improved ASL height and MCT rate, which were significantly greater than improvements observed with ETI alone, demonstrating the benefits of the dual approach.

**Conclusions:**

Selective furin inhibition has the potential to further improve clinical outcomes for all people with CF and offers opportunity as an adjunct to improve responses to currently available CFTR modulator therapies.

## Shareable abstract

**Selective furin inhibition by BOS-318 robustly inhibits ENaC activity and enhances ETI-mediated improvements in airway hydration status as shown by the significant improvements observed in both airway surface liquid height and mucociliary transport** https://bit.ly/3TF71If

## Introduction

Cystic fibrosis (CF) is an autosomal recessive condition that is caused by loss-of-function mutations in the CF transmembrane conductance regulator (*CFTR*) gene [1]. *CFTR* encodes a cAMP-dependent Cl^−^ transporter, the correct functioning of which helps to maintain effective airway mucociliary clearance (MCC) [2]. In CF lung disease, a reduction in both the quality and quantity of CFTR causes airways dehydration, impaired MCC and mucus obstruction. This predisposes individuals to chronic cycles of infection and inflammation, leading to lung injury and a progressive decline in pulmonary function [1].

The approval of CFTR modulator drugs by the European Union and United States, targeting the basic defect in CF has reduced treatment burden and transformed the clinical management of people with CF [3]. Of note, triple CFTR modulator therapy, elexacaftor–tezacaftor–ivacaftor (Trikafta/Kaftrio®, Vertex Pharmaceuticals, Boston, MA, USA; ETI) has been shown to correct CFTR function to ∼62% of wild-type (WT) in nasal epithelial cells [4] and increases CFTR activity to ∼40–45% of WT in F508del homozygous patients, as measured by nasal potential difference [5]. Clinically, ETI shows improvements in lung function and a reduction in exacerbations, hospitalisations, treatment burden and lung transplant referrals [6–8].

For the majority of patients with chronic CF lung disease, the improvement in pulmonary function, however, does not typically reach the normal range [6]. Therefore, exploring new treatment options to augment ETI-mediated airway hydration and MCC holds the promise of additional clinical benefit.

An alternative and long-standing treatment strategy is to target the epithelial sodium channel (ENaC) [9]. ENaC-mediated Na^+^ absorption causes the movement of water away from the apical surface of the airway epithelium, and in the absence of CFTR, contributes to airway surface dehydration, mucous stasis and the collapse of effective MCC, a key contributor to the pathogenesis of CF in the lungs [10]. ENaC has a trimeric structure consisting of α, β and γ subunits that require proteolytic activation, without which the channel only exhibits low conductance for Na^+^ [11]. Furin, a cellular, ubiquitously expressed proprotein convertase cleaves two sites on the α subunit and one on the γ subunit to prime a distinct pool of ENaC as it passes through the biosynthetic pathway [11, 12]. The activation of ENaC is completed at the cell surface by the action of other channel-activating proteases such as prostasin and neutrophil elastase [13–15].

We have previously demonstrated that BOS-318, a cell-permeable and highly selective potent inhibitor of furin robustly suppresses ENaC activity and improves airway surface hydration status and mucociliary transport (MCT) in a fully differentiated CF airway epithelial cell model [16]. In the current study, our aim was to determine whether furin-mediated ENaC inhibition offers an opportunity to enhance ETI-driven airway hydration and MCT, which could provide additional benefits beyond those achieved with ETI alone.

## Materials and methods

### Reagents

All chemicals were obtained from Merck (Rahway, NJ, USA) unless indicated otherwise. CFTR modulators (VX-661 and VX-770) were from Adooq Biosciences (Irvine, CA, USA). VX-445 was synthesised by ChemScene (Monmouth Junction, NJ, USA). BOS-318, previously reported as a highly selective inhibitor of furin [16], was provided by Boston Pharmaceuticals (Boston, MA, USA). Dextran tetramethylrhodamine and FluoSpheres Polystyrene Microspheres (1.0 µm, yellow–green, fluorescent) were sourced from Thermo Fisher Scientific (Horsham, UK). Ultroser G was from CytoGen GMBH, Greven, Germany(. Bovine brain extract and transferrin were from Lonza (Slough, UK). HyClone Fetal clone II was from Scientific Laboratory Supplies Systems (Nottingham, UK).

### Primary human bronchial epithelial cells

HBECs used in this study were derived from donors homozygous for the F508*del* mutation (obtained as a kind gift from R. Bridges, Rosalind Franklin University of Medicine and Science, North Chicago, IL, USA and obtained under institutional review board-approved protocols) or purchased from Epithelix (Geneva, Switzerland), Switzerland. The HBEC culture conditions have been described in detail previously [17]. Briefly, cells were differentiated by plating on Transwell filters, then grown at an air–liquid interface using HBEC differentiation medium (DMEM/F12 supplemented with 2% (v/v) Ultroser G, 2% (v/v) HyClone Fetal Clone II, 0.25% (v/v) bovine brain extract, 20 nM hydrocortisone, 500 nM triiodothyronine, 250 nM ethanolamine, 1.5 µM epinephrine, 250 nM phosphoethanolamine, 2.5 µg·mL^−1^ transferrin, 2.5 µg·mL^−1^ insulin and 10 nM retinoic acid) for at least 4 weeks resulting in a mucociliary phenotype.

### Treatment of CF HBECs with CFTR modulators and BOS-318

CFTR modulator compounds were added to the basolateral compartment at the following doses: VX-445 3 µM, VX-661 18 µM and VX-770 1 µM as previously described [18]. BOS-318 (0.3 µM) was also applied basolaterally. All CFTR modulators and BOS-318 used in the study were prepared in dimethylsulfoxide (DMSO). Cells were treated with equal doses of DMSO in all experiments. In experiments involving ASL height and MCT rate determination experiments, treatments were performed in the presence of 30 nM vasoactive intestinal peptide (VIP), to stimulate CFTR activity, as previously described [19].

### Electrophysiological analysis

For long-term treatments, test compounds were applied to the basolateral compartment of differentiated HBECs for a period of 48 h. For experimentation, cells were switched from differentiation medium to the following bath solution, HEPES-buffered (pH 7.4) F12 Coon's modification medium (Merck, F6636), which was applied apically and basolaterally at 37°C (without CO_2_) for an equilibration period prior to an experimental run. To determine the equivalent current (I_eq_), the transepithelial voltage (V_t_) and resistance (R_t_) were recorded with a 24-channel TECC robotic system (EP Devices, Bertem, Belgium) and I_eq_ calculated using Ohm's law (I_eq_=V_t_*/*R_t_). The measured V_t_ and R_t_ values were corrected for the electrode offset potential, and series resistance of the solution/blank Transwell filter, respectively, at each time point. Amiloride was used to block ENaC activity and forskolin was applied to activate CFTR *via* a cAMP-dependent mechanism. The CFTR-selective inhibitor CFTRinh-172 was used to determine the relative contribution of CFTR *versus* alternate forskolin-activated ion transport pathways. Finally, UTP was included as an index of calcium-activated chloride channel activity. By convention, a positive deflection in I_eq_ is defined as a net movement of anions in the basolateral to apical direction.

### Confocal microscopy measurement of airways surface liquid height

Determination of HBEC airway surface liquid (ASL) height has been reported previously [17]. Briefly, CF HBECs were washed apically with sterile PBS to remove excess surface mucus routinely during culture and before experimentation. Basolateral medium was replaced with fresh medium containing the relevant compound treatments. The ASL layer was labelled with 10 µL PBS containing 2 mg·mL^−1^ of dextran tetramethylrhodamine (10 kD). After an incubation period of 48 h ASL height was measured *via* acquisition of high resolution XZ images (Leica SP8 confocal microscope). During image acquisition, PFC-77 was added apically to prevent evaporation of the ASL layer. To measure the average height of the ASL 10 pre-determined points configured in a spiral fashion, originating at the centre of the culture, were XZ scanned. Data analysis was performed using Image-Pro Premier (Media Cybernetics, Rockville, MD, USA) image analysis software.

### Measurement of mucociliary transport rate in HBECs

Differentiated CF HBECs were washed apically with sterile PBS to remove excess surface mucus routinely during culture and before experimentation. Basolateral medium was replaced with fresh medium containing the relevant compound treatments and 20 µl PBS containing a 1:10 000 dilution of polystyrene FluoSpheres (1 µm yellow–-green microspheres) added apically. Mucociliary transport (MCT) rate measurements were performed 48 h later by capturing 10-s videos of microsphere movement using a Nikon 6D Live-Cell Imaging Microscope. Cells were maintained at 37°C and 5% CO_2_ throughout video capture. Microsphere tracking was performed using the Image-Pro Premier (Media Cybernetics) image analysis software platform to determine the velocity of movement in µm·s^−1^.

### Data analysis and statistics

Data analysis was performed using GraphPad Prism software (version 8) unless indicated otherwise. Normality testing was performed using the D'Agostino and Pearson test. Comparisons between two groups was determined using a Mann–Whitney U-test. Comparisons between three or more groups was determined using the nonparametric Kruskal–Wallis test with Dunn's multiple comparison *post hoc* test. A p-value <0.05 was taken to be significant. All graphs presented represent mean±sem unless otherwise stated.

## Results

### Effect of BOS-318 treatment in combination with ETI on ion channel function

**FIGURE 1:**
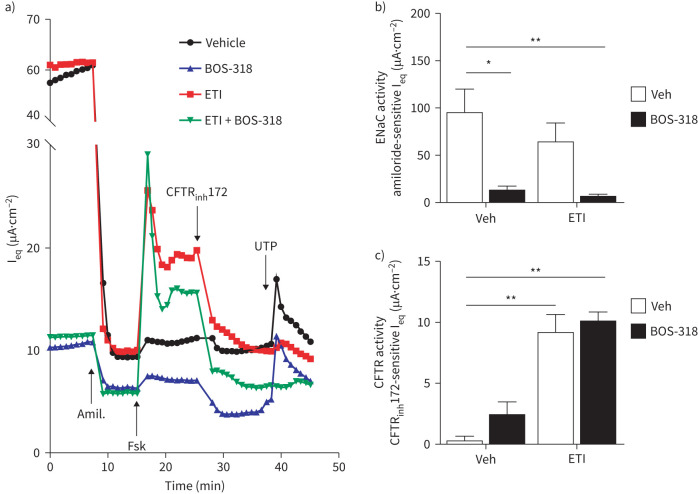
**Effect of long-term BOS-318 treatment in combination with ETI on ion channel function.** Cystic fibrosis human bronchial epithelial cells were treated with either vehicle or BOS-318 in the presence or absence of elexacaftor–tezacaftor–ivacaftor (ETI) for 48 h. After the treatment period, baseline I_eq_ was established, followed by the sequential addition of 10 µM amiloride, 20 µM forskolin, 20 µM CFTRinh-172 and 100 µM UTP. (a) Representative equivalent current (I_eq_) recordings. (b) CFTRinh-172-sensitive I_eq_ representing CFTR activity. (c) Amiloride-sensitive I_eq_, representing epithelial sodium channel (ENaC) activity. n≥5 individual inserts from three donors. Statistical analyses were performed using a Kruskal–Wallis test and Dunn's multiple comparisons test in comparison to the relevant dimethylsulfoxide control. *: p≤0.05; **: p≤0.01. CFTR: cystic fibrosis transmembrane conductance regulator.

CF HBECs were treated with both ETI and BOS-318 (at a dose of 0.3 µM) for 48 h (singly and in combination) and their effect on ion channel function assessed (figure 1). Treatment of CF HBECs with ETI alone showed a significant increase in CFTR-mediated Cl^−^ secretion (p≤0.01) (figure 1b). A modest increase of CFTR-mediated Cl^−^ secretion, was also observed with BOS-318 alone, with equivalent current (I_eq_) increasing from a mean value of 0.33 µA·cm^−2^ in vehicle-treated CF HBECs, to a mean value of 2.46 µA·cm^−2^; however, the increase was not statistically significant (p=0.79). In combination, the inclusion of BOS-318 had no significant effect on the ability of ETI to stimulate CFTR activity (figure 1b). In figure 1c, and in keeping with our previously reported study [16], we show a significant inhibition of ENaC by BOS-318, which was maintained in the presence of ETI (p≤0.01). ETI treatment alone showed a modest, but nonsignificant inhibition of ENaC activity (figure 1c). We also note that the activity of the calcium-activated chloride channel, TMEM16A, stimulated by the addition of purinergic receptor antagonist UTP, was abrogated by ETI but not by BOS-318 (figure 1a).

### The combination of BOS-318 and ETI augments ASL height in CF HBECs compared with ETI alone

**FIGURE 2:**
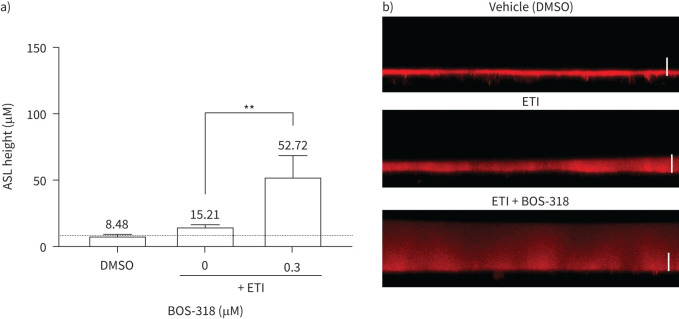
**BOS-318 treatment enhances ETI-mediated improvements in airways hydration as measured by ASL height.** Cystic fibrosis human bronchial epithelial cells were treated with vehicle, elexacaftor–tezacaftor–ivacaftor (ETI) or a combination of ETI and BOS-318, in the presence of 30 nM vasoactive intestinal peptide, for 48 h. (a) Airway surface liquid (ASL) height measurements performed using confocal microscopy. n≥5 individual inserts from two donors. Statistical analyses were performed using a Mann–Whitney U-test. **: p≤0.01. (b) Representative (×40) confocal microscopy images, captured in the XZ plane. The white scale bar represents an ASL height of 20 µm. DMSO: dimethylsulfoxide.

Next, we wanted to determine the impact of ETI-rescued CFTR activity and concurrent BOS-318-mediated ENaC suppression on airway hydration. In keeping with other reports, treatment of CF HBECs by ETI (alone) significantly increased ASL height (in our experiments, from a mean value of 8.48 µm to 15.21 µm, representing a nearly twofold increase) (figure 2a). As reported in our previous study, BOS-318-only treatment led to a ∼35% increase in ASL height in CF HBECs (from a mean of 8.02 to 10.87 μm) [16]. In combination, ETI and BOS-318 were able to increase ASL height to a level that was 3.5-times that observed for ETI alone (mean 52.72 µm) (figure 2a). Representative images demonstrating the large ASL volumes observed on the surface of the differentiated HBEC cultures are shown in figure 2b and supplementary figure 1.

### BOS-318 treatment enhances ETI-mediated improvements in mucociliary transport

**FIGURE 3:**
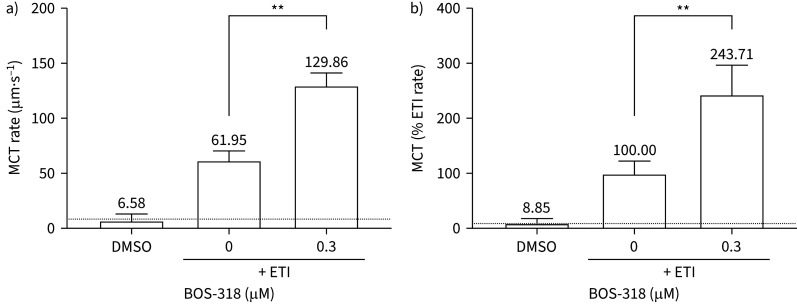
**BOS-318 treatment enhances ETI-mediated improvements in MCT.** Cystic fibrosis human bronchial epithelial cells were treated with vehicle, BOS-318, elexacaftor–tezacaftor–ivacaftor (ETI) or a combination of ETI and BOS-318, in the presence of 30 nM vasoactive intestinal peptide, for 48 h. The mucociliary transport (MCT) rate was determined by tracking fluorescently labelled microspheres on the apical surface of the cells. (a) MCT rate shown as μm·s^−1^ and (b) MCT rate shown as a % of triple CFTR modulator therapy rate. n=7 individual inserts from four cystic fibrosis donors. Statistical analyses were performed using a Mann–Whitney U-test. **: p≤0.01. CFTR: CF transmembrane conductance regulator; DMSO: dimethylsulfoxide.

We wanted to determine whether the increases in ASL height observed when CF HBECs were treated with a combination of BOS-318 and ETI would translate to improved MCT, by tracking the velocity of movement of apically applied fluorescent microspheres. We previously observed a considerable (∼30-fold) increase in the MCT rate (from a mean value of 0.88 to 25.50 μm·s^−1^) when CF HBECs were treated with BOS-318 only [16]. Consistent with the ion channel function and ASL height studies, treatment with ETI caused an increase in MCT rate from a mean value of 6.58 µm·s^−1^ to 61.95 µm·s^−1^ (figure 3a), an almost 10-fold increase. When BOS-318 and ETI were combined, a further approximately 2.5-fold increase in the MCT rate was evident compared with CFTR triple-modulator therapy alone (figure 3a,b; p≤0.01), confirming that ENaC inhibition enhances the effect of CFTR modulators on MCT. Representative captured videos of the MCT experiments are available in the online supplementary material (supplementary videos 1–3).

## Discussion

The US Food and Drug Administration-approved triple CFTR modulator therapy, ETI has shown significant therapeutic benefit in people with CF, with an approximate 10–14% improvement in percentage predicted forced expiratory volume in 1 s (FEV_1_ % pred) and a 60% reduction in exacerbations [6, 20]. ETI therapy, however, does not restore CFTR function to WT levels and is not curative [4, 5]. There is, therefore, a rationale to pursue other treatments that may provide clinical benefit in CF when used as an adjunct to existing CFTR modulator regimens. We report that fully differentiated CF bronchial epithelial cells treated with ETI in combination with a highly selective furin inhibitor, BOS-318, showed improved CFTR-mediated Cl^−^ secretion and a concurrent reduction in ENaC-mediated Na^+^ absorption. Together these changes in ion channel function resulted in significantly improved ASL height and MCT, above the levels achieved with ETI treatment alone.

The potential for ENaC inhibition to be used in combination with CFTR modulation to improve patient outcomes has been previously discussed [9]. It is well established that ENaC is a key regulator of airway hydration and that inhibition causes hyperpolarisation of the apical airway epithelial membrane, creating an increased electrical driving force and thereby inducing chloride secretion [21]. The possibility of an additive effect between ENaC inhibition and CFTR modulation was also reported when ENaC inhibitor BI 1265162 was used alongside ivacaftor–lumacaftor [22]. To date, however, traditional small-molecule direct ENaC blockers such as amiloride, benzamil, GS-9411, BI 443651, BI 1265162 and VX-371 and the peptide analogue SPX-101, although generally well tolerated, have failed clinical development. Reasons have included lack of potency and/or short half-life, dosing issues, efficacy, induced hyperkalaemia, short study duration, and for those that made phase II, lack of end-point sensitivity [9, 23]. Lessons learnt have, however, led to the development of ETD001 (Enterprise Therapeutics, Brighton, UK), a novel inhaled, long-acting direct ENaC blocker that is expected to provide a superior efficacy and safety profile compared with the previous failed compounds. ETD001 has successfully completed phase I safety studies and is being progressed to phase IIa in 2024 [24]. An alternative approach to ENaC inhibition is, however, offered by BOS-318 and other compounds within the patent family (*e.g.* BOS-857 and BOS-981) [16, 25]. These compounds are first-in-class, highly selective and potent inhibitors of furin, which has been found to be elevated in CF airway epithelial cells and is the prime protease responsible for ENaC activation [16, 17, 26]. In a follow-up to our previous report that discussed the development of BOS-318, protection against neutrophil elastase (NE)-mediated activation of the ENaC and the effect of its robust inhibition of ENaC on ASL height and MCT [16], we now report that BOS-318 augments ETI treatment by further improving the hydration status of the airway epithelium leading to a significant increase in MCT. The ability of BOS-318 to further uplift these parameters in our *ex vivo* models has the potential to compensate for the inability of ETI to fully normalise CFTR activity to WT levels [4, 5]. In the future, benchmarking against MCT observed in non-CF cells and *in vivo* investigations, potentially involving a direct comparison with the current best-in-class ENaC blocker, ETD001 will offer valuable insights into the utility of BOS compounds as clinical candidates.

The broader advantages of furin inhibition in CF lung disease compared with direct ENaC channel blockade are also being explored. Beyond its impact on ENaC, furin plays a pivotal role in activating numerous other cellular substrates (bacterial and viral, as well as mammalian), several of which are crucial to the pathogenesis of CF airways disease [25]. Examples include furin activation of *Pseudomonas aeruginosa* exotoxin A (PEA), a toxic product of *P.* *aeruginosa* infection, which is a major cause of morbidity and mortality in people with CF [27]. Of note, treatment of CuFi cells with BOS-318 provided cytoprotection against toxicity induced by PEA (median effective concentration of 47.8 nM) [16]. BOS inhibitors (BOS-318, BOS-857 and BOS-981) have also been able to effectively block the processing of the secretory type-I membrane-bound spike (S) protein of SARS-CoV-2 in HeLa cells, and also completely prevented SARS-CoV-2 infection of lung-derived Calu-3 cells in combination with camostat, an inhibitor of the type II transmembrane serine protease 2 (TMPRSS2) [28]. Another substrate regulated by furin is transforming growth factor-β (TGF-β), which is found in elevated concentrations in bronchoalveolar lavage from CF patients [29] and is associated with a reduction in pulmonary function [30]. TGF-β has also been associated with reduced chloride secretion [31] and reduced CFTR protein expression [32] and was also found to disrupt CFTR correction by VX-809 (lumacaftor) [33]. These findings, therefore, suggest the possibility of potentially broader benefits, beyond that of improved airway hydration status and restored MCC, through the combined use of CFTR modulation and furin inhibition.

MCC in the airways can be influenced both by the rate of ciliary beating and the hydration status of the ASL layer, with increased mucus hydration translating to increased mucus velocity [34]. The substantive increase in ASL height observed when BOS-318 and ETI were added in combination was stark (figure 2). Interestingly, studies in individuals with systemic pseudo-hypoaldosteronism who have loss-of-function mutations in the genes for ENaC subunits and fail to absorb liquid from the airway surfaces exhibit high rates of MCC [35]. Moreover, *in vitro* investigations have demonstrated that the introduction of fluid onto airway surfaces results in a selective penetration into the mucus layer. This induces a swelling effect, preserving the connection between the cilia and mucus. Consequently, the addition of liquid has been observed to enhance mucus transport rates [36].

TMEM16A-mediated Cl^−^ secretion has been investigated as a potential compensatory route for loss of CFTR activity in the airway epithelium [37], though there is some debate as to the potential benefits and drawbacks of such as approach [38]. While not the focus of the current study, we did observe that UTP-stimulated TMEM16A activity was lost in the presence of ETI but not by BOS-318 (figure 1a). The phenomenon has been reported previously [39], though the underlying molecular mechanisms explaining this effect are unknown.

Overall, the data presented here demonstrate that furin inhibition by BOS-318 robustly inhibits ENaC activity and enhances ETI-mediated improvements in airways hydration status, as measured by both ASL height and MCT. Highly selective inhibition of furin may therefore, provide an alternative approach to treatment, which, when used with current CFTR modulator regimes, has the potential to deliver an increase in clinical benefit for people with CF.

## Supplementary material

10.1183/23120541.00445-2024.Supp1 **Please note:** supplementary material is not edited by the Editorial Office, and is uploaded as it has been supplied by the author.
Supplementary material 00445-2024.SUPPLEMENT
Video S1 00445-2024.SUPPLEMENT
Video S2 00445-2024.SUPPLEMENT2
Video S3 00445-2024.SUPPLEMENT3

## Data Availability

The authors declare that all data supporting the findings of this study are available within the paper and its supplemental information files.
