# Supplementary material for: BOS-318 treatment enhances elexacaftor–tezacaftor–ivacaftor-mediated improvements in airway hydration and mucociliary transport
Source: ERJ Open Res. 2025 Feb 25;11(1):00445-2024. doi: 10.1183/23120541.00445-2024 (PMC11863070; doi:10.1183/23120541.00445-2024)
Supplement: Supplementary file 1 [file 00445-2024.SUPPLEMENT.pdf]

BOS-318 treatment enhances elexacaftor-tezacaftor- ivacaftor (ETI)-mediated improvements in airways hydration and mucociliary transport.

Lisa E.J. Douglas[1], James A. Reihill[1], S. Lorraine Martin[1*]

Supplementary Information

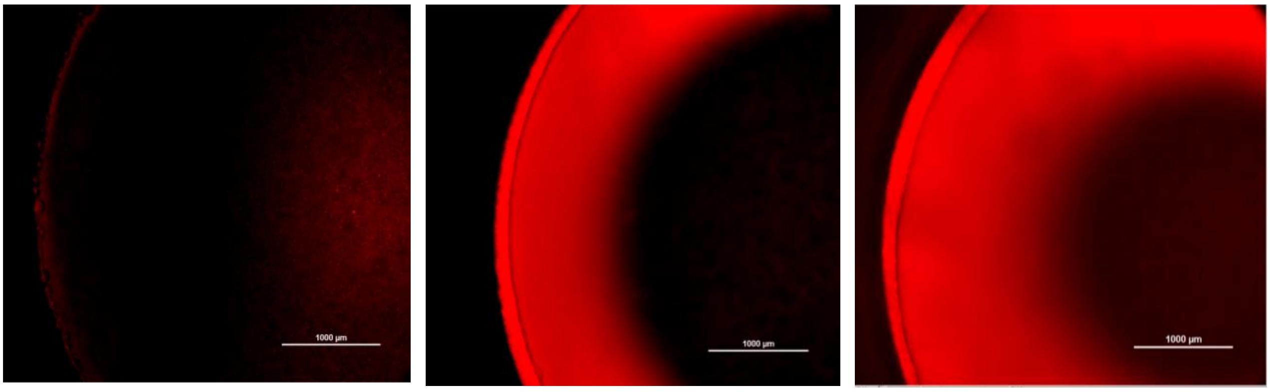

Vehicle **ETI** **ETI + BOS-318**

Supplemental Figure S1 (Related to Figure 2): Representative microscopy images (4X magnification) captured using a Nikon Eclipse Ti inverted microscope showing the ASL menisci. CF HBECs were treated for 48 hours with either vehicle, ETI (VX-445 3 µM, VX-661 18 µM, VX-770 1µM) or a combination of BOS-318 at a dose of 0.3 µM with ETI. Treatments were added in the presence of 30 nM vasoactive intestinal peptide (VIP). Dextran Tetramethylrhodamine 10,000 MW was added at the time of treatment to the apical side to label the airway surface liquid.

Supplemental Movie Files (Related to Figure 3): Representative videos captured at 10X magnification demonstrating MCT rate as determined by the movement of apically applied fluorescent microbeads on the surface of CF HBECs. CF HBECs were treated for 48 hours with either **(1)** vehicle (DMSO), **(2)** ETI (VX-445 3 µM, VX-661 18 µM and VX-770 1 µM) or **(3)** ETI + BOS-318 0.3 µM.

Supplementary Files

Video S1: Vehicle (DMSO) – *Douglasetal_10X_Vehicle.mp4*

Video S2: ETI– *Douglasetal_10X_ ETI.mp4*

Video S3: ETI + BOS-318 (0.3 µM) – *Douglasetal_10X_ETI+BOS-318_0.3uM.mp4*
